# Immunohistochemical staining of LEF-1 is a useful marker for distinguishing WNT-activated medulloblastomas

**DOI:** 10.1186/s13000-022-01250-3

**Published:** 2022-09-13

**Authors:** Depeng Wang, Jie Gong, Hui Zhang, Yulu Liu, Nannan Sun, Xiaomeng Hao, Kun Mu

**Affiliations:** 1 Department of Pathology, School of Basic Medical Sciences, Shandong University, Jinan, 250012 Shandong China; 2 Department of Neurosurgery, Qilu Hospital of Shandong University and Institute of Brain and Brain-Inspired Science, Shandong University, Jinan, 250012 Shandong China; 3 Shandong Key Laboratory of Brain Function Remodeling, Jinan, 250012 Shandong China; 4 Department of Pathology, Qilu Hospital of Shandong University, Jinan, 250012 Shandong China

**Keywords:** LEF-1, β-catenin, Immunohistochemistry, WNT-activated, Medulloblastoma

## Abstract

**Objectives:**

To investigate lymphoid enhancer factor 1 (LEF-1) protein expression in medulloblastomas (MBs) and its correlation with molecular grouping of MBs.

**Methods:**

Expressions of LEF-1 and β-catenin were detected by immunohistochemistry, and molecular grouping was performed based on the NanoString and sequencing techniques for 30 MBs.

**Results:**

By genetic defining, 3 MBs were WNT-activated, 11 were SHH-activated, 3 were in Group 3 and 13 in Group 4 respectively. Nuclear LEF-1 staining was found in 8 MBs using immunohistochemical method. Three out of 8 showed diffuse and strong nuclear LEF-1 staining which were proved to be WNT-activated genetically, while the other 5 MBs with focal staining were SHH-activated genetically. The expression of LEF-1 protein was significantly correlated with genetically defined WNT-activated MBs (*P* < 0.0001). We also found focal nuclear β-catenin expression ( less than 1% of tumor cells) in 5 MBs. LEF-1 positivity was significantly correlated nuclear β-catenin expression (*p* < 0.001).

**Conclusions:**

Immunohistochemical staining of LEF-1 can be used as a supplement for β-catenin to diagnosis WNT-activated Medulloblastomas, when β-catenin is difficult to recognize for its cytoplasm/membrane staining background. Diffuse nuclear staining of LEF-1 indicates WNT-activated MB.

## Background

Medulloblastoma (MB) is the most common malignant embryonal tumor of the central nervous system in childhood [1]. It presents with high potential of metastases and poor prognosis. The 5-year survival rate has increased from 20% in 1970 to 65–70% nowadays with the development of new treatment methods for MB [2]. However, excessive radiotherapy and chemotherapy bring serious sequelae on children, including impairment of language and cognitive function, serious decline of IQ, hearing loss and so on [3]. It is urgent to explore how to select suitable patients and reduce toxicity without sacrificing survival benefit. MB is a highly heterogeneous tumor which can be divided into four morphological variants: classic, desmoplastic/nodular, anaplastic/large cell and medulloblastoma with extensive nodularity (MBEN) [4]. However, histological variants fail to completely correspond with clinical manifestations. More importantly, morphology alone is difficult to predict clinical prognosis.

According to the molecular characteristics of the fifth edition of the WHO Classification of Tumors of the Central Nervous System (CNS), published in 2021, medulloblastomas are defined into four groups: WNT-activated, SHH-activated and TP53-wildtype,SHH-activated and TP53-mutant and non-WNT/non-SHH [5]. Each subgroup not only has unique population genetics and clinical manifestations, but also has a certain predictive effect on prognosis and treatment sensitivity [3, 6]. WNT-activated MB account for about 10% of the total, and most of them are found in children aged 6–10 years which has the highest sensitivity to postoperative chemoradiotherapy, and is the least likely to metastasize [7]. Nearly 90% of the WNT-activated MB patients survive for more than 5 years, and the 10-year survival rate of children can reach 95% [8]. Therefore, it is of great clinical significance to establish a specific and sensitive identification method suitable for the current pathological laboratory to choose WNT-activated MB patients with an improved treatment scheme and reduce the treatment sequelae.

CTNNB1gene mutation (more than 90%) is the main genetic variations of the WNT-activated MBs which results in the amino acid residues change at the phosphorylation site of β-catenin [9, 10]. Therefore, the somatic activating mutations in exon3 of CTNNB1 (encoding β-catenin) can cause stabilization of β-catenin, which lead to β-catenin accumulation in the nucleus.Theoretically, the immunohistochemical accumulation of β-catenin in the nucleus can be used to identify WNT-activated MBs. However, in the routine pathological work, we encountered the low sensitivity of β-catenin immunohistochemical staining. Moreover, no standardized criteria for interpretation of β-catenin immunostaining was reached. Interpretation of β-catenin staining must be performed carefully considering its background cytoplasmic or membranous staining. β-catenin interacts specifically with lymphoid enhancer factor 1 (LEF-1)in the nucleus to form a transcription complex, which regulates Wnt signaling control the expression of downstream target genes involved in cell cycle control, such as c-Myc and Cyclin D1. The specific interaction between β-catenin and LEF-1 led us to infer LEF-1 might be used as identifying marker for WNT-activated MBs. Thus, in the current study, we examined the immunohistochemistry expression of LEF-1 in MBs, and compared its value with β-catenin to predict Wnt-activated MBs.

## Materials and methods

### Patients

A total of 30 formalin-fixed paraffin-embedded (FFPE) medulloblastomas samples were collected from the Department of Pathology, Qilu Hospital of Shandong University of China from 2018 to 2020. The H&E stained slides were reviewed independently by two pathologists blind to the clinical information to confirm the pathological diagnosis, and further defined the tumors to four histological subgroups as classic, desmoplastic/nodular, anaplastic/large cell or medulloblastoma with extensive nodularity (MBEN) according to the WHO 2016 classification of tumors of Central Nervous System. All protocols follow the ethical guidelines of the Helsinki Declaration and were approved by Shandong University Research Ethics Committee.

### Immunohistochemistry

Thirty formalin-fixed paraffin-embedded (FFPE) from selected MB tumor samples fixed in 10% neutral buffered formalin were retrieved for immunohistochemistry. Samples from case of desmoid-type fifibromatosis confirmed by CTNNB1 3 exon mutation and small lymphocytic lymphoma as positive control for β-catenin and LEF-1 respectively. After deparaffinization, sides were subjected to antigen retrieval by autoclave in 0.01 M EDTA buffer (pH 8.5) for 3 min 15 s, followed by incubation in 3% H2O2 for 10 min to quench endogenous peroxidase. Sections were incubated overnight at 4 °C with monoclonal antibody LEF-1 (clone EP310, ZSGB-BIO, China, ready-to-use) and β-catenin (clone UMAB15, ZSGB-BIO, China, ready-to-use). Substitution of the primary antibody with PBS was served as a negative control. Slides were washed with PBS for 3 times and incubated with secondary antibody (PV9000, ZSGB-BIO, Beijing, China) at room temperature for 0.5 h. Staining was carried out by incubating the slides in 3, 3′-diaminobenzidine (DAB), followed by counterstained with hematoxylin, dehydrated in gradient ethanol and xylene. LEF-1 was localized in the nuclei of tumor cells. Its expression level was analyzed through assessing the percentage of stained tumor cells as follows: sections with no staining cells were regarded as negative, 1–50% positive cells as partial positive and 51–100% positive cells as diffuse positive. Any nuclear staining of β-catenin was defined as positive regardless of the percentage of stained tumor cells.

### Whole exome sequencing

Tumor DNA was purified from 5–10 pieces of 5 μm slides from FFPE blocks using QIAamp DNA FFPE Tissue Kit (Qiagen) following the manufacturer´s instruction. DNA was quantified with the Qubit Fluorometer V2.0 using the dsDNA Assay Kit (Thermo Scientific). DNA samples with a total amount greater than 100 ng were selected to perform whole exome sequencing.

Whole genome DNA-sequencing libraries were constructed using the Rapid DNA Lib Prep Kit for Illumina(Abclonal)following the manufacturer’s guidelines. In brief, Genomic DNA was randomly fragmented to segments of 350 to 500 bp by Covaris sonication, end-repaired, A-tailed, adaptor ligated and PCR amplified followed by a quality control step using Agilent 4200 TapeStation System. Then, qualified samples were hybridized to KAPA HyperExome probes(Roche) to enrich human exome with KAPA HyperCapture Reagent kit (Roche). 150 base paired-end sequencing was performed on the Illumina Novaseq 6000.

### DNA sequencing data processing

Sequenced Reads were aligned to the Human Genome Reference Consortium build 37 (GRCh37) using BWA-mem v0.7.16-r1188 with default settings. Then, SAMtools v1.9 and Picard tools v1.125 (Broad Institute) were used to sort the reads by coordinates and to mark the duplicates. Reads with a phred-scaled base quality below 20 were removed. The CNVkit v0.9.6 was used to estimate the status of copy number variation with default parameters. Varscan2 v2.4.1 was used to call SNVs and Indels. The filter procedures were performed as recommended and filter thresholds were set as default to identify high quality SNVs and indels. The vcf calls were then annotated using multiple publicly available tracks such as 1000 Genome variants, single nucleotide polymorphism database (dbSNP), COSMIC, Clinvar and other elements. The functional effect of the mutations was annotated using Annovar. Owing to the poor coverage of the TERT promoter region, TERT promoter mutation was tested by Sanger sequencing.

Machine learning algorithm was utilized to build the MB classification model. Training sets derived from the combination of ICGC medulloblastoma cohort and own in-house data. Random Forest algorithm was used to build a probability model to calculate the probability of a given medulloblastoma sample belonging to one of 4 subtypes. Features used in this process included somatic mutation and copy number alteration (CNA). the number of chromosome level CNV. Besides, clinical features such as gender, age and histological defined subtype were also included in the classification model.

### NanoString analysis

Molecular subtyping based on the NanoString was performed for 5 MBs. Total RNA was purified from FFPE blocks using a RNeasy FFPE Kit (Qiagen) following the manufacturer´s instruction. RNA concentration was measured, and RNA integrity assessed using the Agilent 2100 bioanalyzer. Reporter Coreset and Capture ProbeSet were overnight hybridized to the total RNA in thermocycler (Applied Biosystem). Post Hybridization processing and data collection for gene expression was done using nCounter Sprint Profiler system, as per manufacturer’s protocol.

### Statistical analysis

Statistical analyses were performed using the SPSS for Windows version 19.0 (SPSS Inc., IL, USA). The relationships between LEF-1 expression and clinicopathological parameters were analyzed using Pearson Chi-square test and Fisher’s exact test. *P*-value < 0.05 was considered statistically significant.

## Results

### Clinicopathologic features

**Fig. 1:**
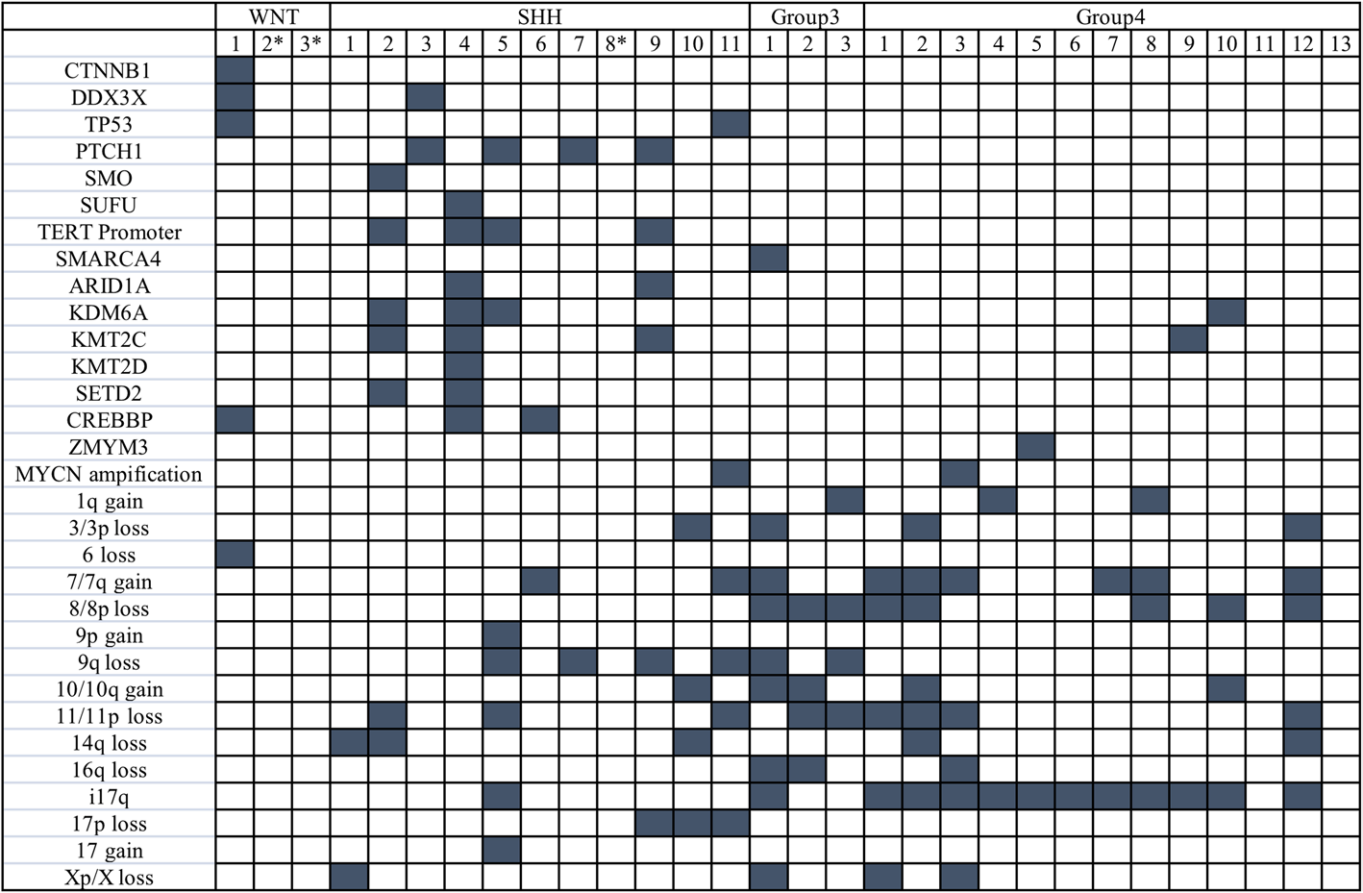
Summary of whole exome sequencing and NanoString results. *The result of molecular typing by NanoString. Navy blue, positive; empty, negative

Two experienced pathologists confirmed the diagnosis of MB for all the enrolled cases with a group of the routine antibodies at least include Syn, CgA, NeuN, GFAP, INI-1, and Ki67 for possible differential diagnosis. Clinicopathologic characteristics of the MBs patients were summarized in Table 1. Histologically, 30 MBs consisted of 17 classic MBs, 7 desmoplastic/nodular MBs, 6 anaplastic/large cell MBs, and 0 MBEN according to the WHO 2016 classification of tumors of Central Nervous System. Genetically, there were 3 cases of WNT-activated, 11 cases of SHH-activated, 3 cases of group3, and 13 cases of group4 MBs respectively (Fig. 1).

**Table 1:** Summary of clinical and histopathologic features of 30 MBs

	No. of Cases (%)
Age at diagnosis Median(range)	12.5 years (0–43) years
≤ 3 years	3(10)
> 3 and ≤ 10 years	11(36.7)
> 10 and ≤ 17 years	8(26.7)
> 17 years	8(26.7)
Gender	
Male	16(53.3)
Female	14(46.7)
Histologically defined	
Classic	17(56.7)
Desmoplastic/nodular	7(23.3)
Anaplastic/large cell	6(20)
Genetically defined	
WNT-activated	3(10)
SHH-activated	11(36.7)
Group3	3(10)
Group4	13(43.3)

### LEF-1 expression

**Fig. 2:**
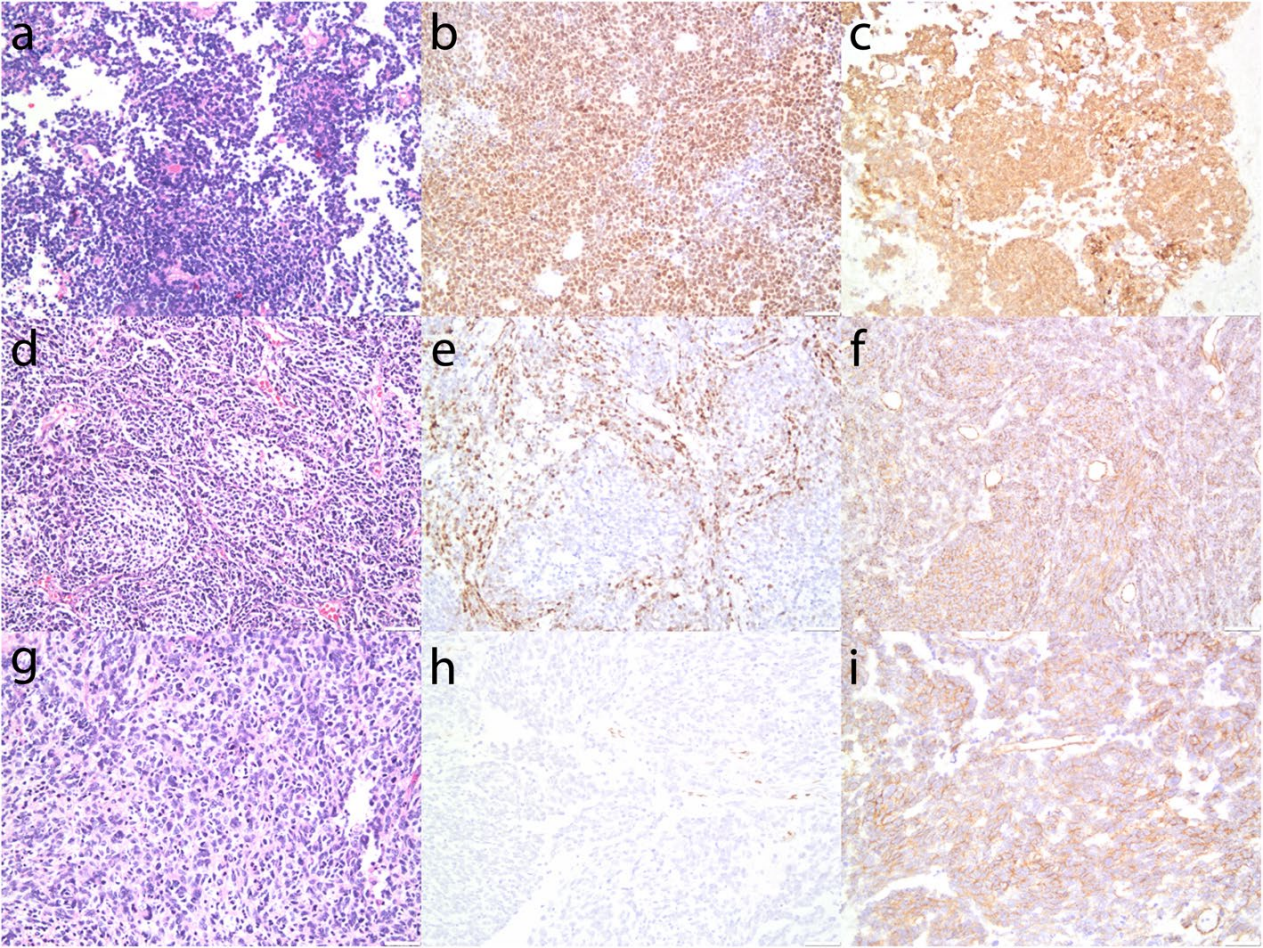
LEF-1 and β-catenin expression pattern in different histological and genetical MB. LEF-1 and β-catenin expression in WNT-activated MB, H&E staining showed classic histological features (**a**), diffuse nuclear LEF-1 expression (**b**), and focal nuclear β-catenin expression (**c**). A case of SHH-activated MB showed desmoplastic/nodular features (**d**, H&E staining), focal LEF-1 (**e**) and no nuclear β-catenin expression (**f**). A case of Group 4 MB showed Anaplastic/large cell features (**g**, H&E staining), no LEF-1 expression (**h**) and no nuclear β-catenin expression (**i**)

Using the clone EP310 antibody, LEF-1 was detected in 8 of 30 (26.7%) MBs (Fig. 2). Among them, 5 (16.7%) cases were partial staining, while 3 (10%) cases showed diffuse strong staining. In these 5 LEF-1 partially expressed MBs, 3 cases were desmoplastic/nodular MBs histologically, one was classic, and one was were anaplastic/large cell MB. All the 5 LEF-1 partially expressed MBs were grouped into SHH-activated subtype. For 3 MBs cases with LEF-1 diffuse and strong expression, all showed classic histological feature, and genetically were WNT-activated MBs (Table 2). WNT-activated MBs correlate significantly with nuclear LEF-1 staining. No correlation was found between LEF-1 expression and histological subgroups.

**Table 2:** Summary of LEF-1 and β-catenin expression relating to histological and molecular subgroups of MBs

	Nuclear β-catenin		*P* value	LEF-1			*P* value
	Yes	No		Diffuse	Focal	Negative	
Histologically defined							
Classic	3	14	0.618	3	1	13	0.186
Desmoplastic/ nodular	0	7		0	3	4	
Anaplastic/ large cell	1	5		0	1	5	
Genetically defined							
WNT-activated	3	0	0.0014*	3	0	0	0.0001*
SHH-activated	1	10		0	5	6	
Group3	0	3		0	0	3	
Group4	0	13		0	0	13	

^*^P value < 0.05 was regarded statistically significant

### β-catenin expression

Detecting the protein expression using the clone UMAB15 antibody, All the MBs cases showed tumor cell membrane/cytoplasm expression of β-catenin. Only four MBs showed focal scattered nuclear staining of β-catenin with percentage of stained tumor cell less than 1% (Fig. 2). Three out of the four β-catenin nuclear expression cases were classified as classic WNT-activated MBs. The other one was molecular SHH-activated and histological anaplastic/large cell MB (Table 2). WNT-activated MBs correlate significantly with nuclear β-catenin staining. No correlation was found between nuclear β-catenin expression and histological subtypes. Our result also confirmed LEF-1 protein expression level was correlated with nuclear expression of β-catenin (*p* < 0.001).

## Discussion

Individualized treatment is the trend of therapeutic regime for patients with medulloblastoma. Previous studies have proved that the four molecular subgroups of medulloblastoma have significantly different characteristics regarding to clinical metastases and prognosis [11].Differential high-throughput gene detection analysis between the four molecular subgroups has made a set of molecular markers that enable genetic classification based on immunohistochemistry [12, 13], the NanoString gene expression assay [14] and DNA methylation arrays [15]. NanoString gene expression assay have several key advantages, including sensitivity, reproducibility, and yield high information content from patient [16], which serve as the gold standard for diagnosis of medulloblastoma. Compared with high-throughput gene detection and molecular detection methods, immunohistochemistry is more time-saving, economical and practical in clinical diagnosis. Researchers are looking for fast and effective immunohistochemical markers to predict the risk of medulloblastoma.

WNT-activated medulloblastomas accounts for about 10% of all MBs. It mainly occurs in older children and adolescents with average age of 10 years old. Histologically, classic variants are most in this group. The 5-year overall survival rate is more than 90%. WNT-activated MBs frequently have deletions of chromosome 6, and more than 90% have CTNNB1 gene mutations. [10] Thus, deletions of chromosome 6 and nuclear β-catenin accumulation, serves as a sensitive and highly specific marker for this subgroup of MBs. β-catenin is the main downstream effector of WNT signaling pathway. Under normal circumstances, the cytoplasmic β-catenin is phosphorylated at specific residues through GSK-3 β, and then degraded by ubiquitin proteasome system. When the coding gene CTNNB1 is mutated, WNT signaling pathway is activated, resulting in the accumulation of β-catenin in the nucleus.

Nuclear β-catenin immunohistochemical stain can be useful in the diagnosis of many tumors including desmoid-type fifibromatosis [17], hepatocellular adenoma [18], and solid-pseudopapillary neoplasm of the pancreas [19], which is also used to identify WNT-activated MB in clinicopathologic diagnosing practice [20]. Nuclear β-catenin staining were classified as WNT-activated MB [20, 21]. So far as we know, there is no agreement on the standard of positive cut-off. Another obstacle of the use of β-catenin staining is its low sensitivity, which affects its application in surgical pathological practice [20, 22].

An early study showed two patterns of immunohistochemical expression of β-catenin in a series of 72 paediatric medulloblastomas, β-catenin extensive nuclear staining (> 50% of the tumor cells) and focal nuclear staining (< 10%). However in their study, only the extensive β-catenin nucleopositivity cases harboured CTNNB1 mutations [23]. In our study, we did not found cases with extensive nuclear staining which might be due to our series is small, with insuffificient numbers of WNT-activated MBs. We only found focal nuclear staining, in fact less than 1% of the tumor cells for all the WNT-activated MBs, which was difficult to recognize in a cytoplasm/membrane staining background. Many studies were consistent with our results, it seems that even a minimal percentage of β-catenin–positive nuclei should be considered significant [24]. And most significantly, the presence of rare Wnt-active cells in non-Wnt MBs may functionally retain the impaired tumorigenic potential of Wnt MB, which implies the antitumorigenic role of Wnt activation in non-Wnt MBs [25].

Another possible explanation might be the different clone of anti‐β-catenin antibody we use. But the antibody we used in our study showed excellence sensitivity in tumors of desmoid‐type fibromatosis and solid pseudopapillary neoplasm of the pancreas in our clinical pathology practice. Yamada’s research showed the sensitivity (ranging from 54 to 96%) and specificity (ranging from 62 to 98%) of nuclear β-catenin in the diagnosis of desmoid‐type fibromatosis were different among three anti‐β-catenin antibodies commonly used. Their work also showed comparable results with immunohistochemistry of β-catenin and LEF1 (sensitivity of 88% and specificity of 76%) [26]. The facts that nuclear β-catenin may be negative and nuclear β-catenin positive tumors may lack CTNNB1 mutations [24, 27, 28], made it recommendable to refer to LEF-1 immunohistochemistry when making the diagnosis of WNT-activated tumors. One study suggested deep, uniform nuclear LEF1 combined with β- catenin immunohistochemical staining could be useful in distinguishing deep penetrating nevi from histologic mimics [29]. Singhi’s work also suggest LEF1, as part of an immunohistochemical panel, can be a useful ancillary stain in the diagnosis of solid-pseudopapillary neoplasms [30].

Lymphoid enhancer-factor 1 (LEF-1), a transcription factor that binds DNA in a sequence-specific manner with β-catenin, but has not been fully studied in MBs. Activation of WNT / β-catenin signaling pathway results in reduced degradation of β-catenin, which leads to increased cytoplasmic β-catenin enter the nucleus, and combined with the nuclear transcription factor TCF/LEF-1 to form a transcription complex and regulate the transcription of downstream target genes, such as C-Myc and Cyclin D1 [31, 32]. In vitro, the two proteins bind in a highly cooperative manner [33]. LEF-1 acts like a nuclear anchor, β-catenin can direct interaction with that, providing a molecular mechanism for the transmission of signals to nuclear, driving tumor formation. In addition, β-catenin strongly enhances transcription by LEF-1 in a chromatin-dependent manner on a minimal enhancer composed of reiterated TCF/LEF-1 binding sites in vitro [34]. Considering the close functional relationship between LEF-1 and β-catenin, we speculate that LEF-1 may be a useful marker predicting WNT-activated MBs. In our study, the expression of LEF-1 was detected by immunohistochemistry and compared with β-catenin. Our results clearly showed that LEF-1 expression was significantly related with WNT-activated MBs. We did not find any LEF-1 expression in non-Wnt/non-SHH MBs. For these 3 LEF-1 diffuse strongly expressed MBs, all showed classic histological feature, and geneticlly defined as WNT-activated MBs, while β-catenin expression was difficult to read, and less than 1% tumor cells showed nuclear β-catenin staining for the 4 positive cases. Thus, immunohistochemical staining of LEF-1 can be used as a supplement for β-catenin to diagnosis WNT-activated Medulloblastomas, when β-catenin is difficult to recognize for its cytoplasm/membrane staining background.

In summary, this is the first report that LEF-1 immunohistochemical stain can be used as a more sensitive marker than β-catenin to predict WNT-activated MBs. Hopefully, these data can make contributions towards distinguishing future patients with WNT-activated MBs which need improved treatment scheme and reduce the treatment sequelae. Beyond clinical applicability, mechanistic questions about the functional role of LEF-1 expression needs further investigation in determining the outcome of MBs.
